# A Review of Time Courses and Predictors of Lipid Changes with Fenofibric Acid-Statin Combination

**DOI:** 10.1007/s10557-012-6394-0

**Published:** 2012-05-17

**Authors:** Theodosios D. Filippatos

**Affiliations:** Department of Internal Medicine, School of Medicine, University of Ioannina, 45110 Ioannina, Greece

**Keywords:** Fenofibric acid, Fibrate, Statin, Simvastatin, Atorvastatin, Rosuvastatin, Triglycerides, High-density lipoprotein cholesterol

## Abstract

Fibrates activate peroxisome proliferator activated receptor α and exert beneficial effects on triglycerides, high-density lipoprotein cholesterol, and low density lipoprotein subspecies. Fenofibric acid (FA) has been studied in a large number of patients with mixed dyslipidemia, combined with a low- or moderate-dose statin. The combination of FA with simvastatin, atorvastatin and rosuvastatin resulted in greater improvement of the overall lipid profile compared with the corresponding statin dose. The long-term efficacy of FA combined with low- or moderate- dose statin has been demonstrated in a wide range of patients, including patients with type 2 diabetes mellitus, metabolic syndrome, or elderly subjects. The FA and statin combination seems to be a reasonable option to further reduce cardiovascular risk in high-risk populations, although trials examining cardiovascular disease events are missing.

## Introduction

Fibrates have been used in the treatment of dyslipidemia for many years. Fibrates exert their effects by activating peroxisome proliferator activated receptor α (PPAR-α) [1]. Fibrates are well known for their beneficial effects on triglycerides (TG), high-density lipoprotein cholesterol (HDL-C), and low-density lipoprotein (LDL) subclass distribution [2, 3]. One of the most used fibrates, fenofibrate, has also shown to improve many other atherosclerosis-related variables, such as high sensitivity C-reactive protein (hsCRP), lipoprotein-associated phospholipase A_2_ (LpPLA_2_), apolipoprotein C-III (apoC-III), and reverse HDL cholesterol transport [1, 4–10].

Fenofibrate is a pro-drug, which requires de-esterification in the liver to fenofibric acid, the active drug, which is then released into the plasma to activate PPAR-α in liver, vascular endothelium, adipocytes, and muscle cells [11, 12]. The newer fibrate formulation, fenofibric acid (FA, Trilipix®, Abbott), that was recently approved by the Food and Drug Administration (FDA), is the choline salt of fenofibric acid. FA is not a pro-drug and does not undergo first-pass hepatic metabolism [13]. FA is manufactured as delayed-release 45 mg and 135 mg capsules and can be taken without regard to meals. Upon multiple dosing, FA plasma levels reach steady state within 8 days [14]. FA is administered once daily [14].

In vitro studies using human liver microsomes indicate that FA is a weak inhibitor of CYP2C8, CYP2C19, and CYP2A6, and a mild to moderate inhibitor of CYP2C9 at therapeutic concentrations [14, 15]. Since they are highly protein-bound, all fibric acid derivatives may increase the anticoagulant effect of coumarin derivatives [16, 17], thus the International Normalized Ratio should be carefully monitored. No clinically significant pharmacokinetic interaction has been observed between FA/fenofibrate and statin administration in humans [18–20]. FA administration should be avoided in patients who have severe renal impairment, and dose reduction is required in patients having mild to moderate renal impairment. No pharmacokinetic studies have been conducted in patients with hepatic impairment. FA has not been investigated in trials in pediatric patients.

The aim of the present review is to describe the effects of FA administration combined with a statin in patients with mixed dyslipidemia and to highlight the long-term maintenance of these effects in a wide range of patients.

## Methods

A PubMed/Scopus search was performed up to September 2011 using combinations of “fenofibric acid” with the following keywords: fibrate, fenofibrate, statin, simvastatin, atorvastatin, rosuvastatin, lipid-lowering medications, adverse effects, side effects, gastrointestinal, transaminases, creatine kinase, myopathy, safety. Randomised controlled trials, original papers, review articles and case reports that provide information regarding the pharmacology, lipid and non-lipid effects, duration of biochemical alterations, and adverse events of FA + statin combination are included in the present review. References of these articles were scrutinised for relevant articles.

## Designs of major fenofibric acid trials

**Fig. 1:**
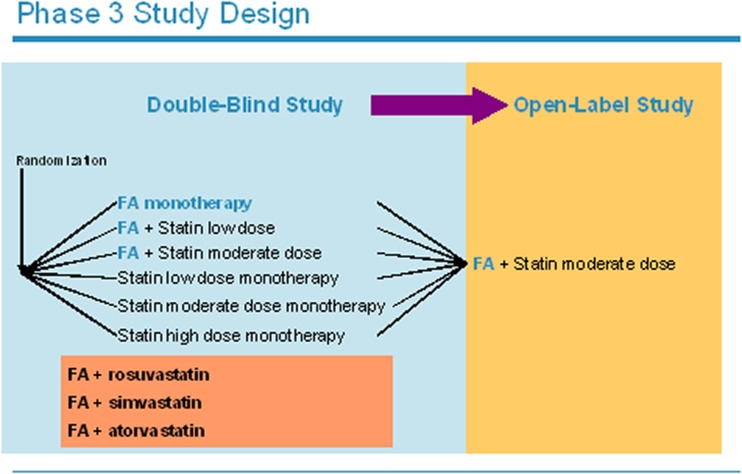
Design of the pivotal trials evaluating the efficacy and safety of the combined use of fenofibric acid (FA) with different statins in patients with mixed dyslipidemia

The efficacy and safety of FA has been evaluated in a large well-designed phase III clinical program consisting of three separate double-blind, randomized, active control trials (Fig. 1) [21]. In each trial patients were randomized in a ratio of 2:2:2:2:2:1 to one of six arms:

- Fenofibric acid 135 mg/day
- Low-dose statin
- Medium-dose statin
- Fenofibric acid 135 mg/day + low-dose statin
- Fenofibric acid 135 mg/day + medium-dose statin
- High-dose statin

A single statin was given in each trial:

- Simvastatin 20, 40 or 80 mg/day [22]
- Atorvastatin 20, 40 or 80 mg/day [23]
- Rosuvastatin 10, 20 or 40 mg/day [24]

Together, these studies investigated the safety and efficacy of FA 135 mg/day as monotherapy and combined with three different statins [21]. The high-dose statin arms were not used for formal statistical comparisons, but served as a clinically relevant reference for assessment of efficacy and safety. They are not described in detail in this review. Primary efficacy endpoints were fasting mean/median percentage changes in HDL-C and TG (comparing each combination with corresponding statin-dose monotherapy) and LDL cholesterol (LDL-C) levels (comparing each combination with FA monotherapy). Inclusion criteria were elevated TG (≥150 mg/dl or 1.69 mmol/L), decreased HDL-C levels (<40 mg/dl or 1.03 mmol/L for men and <50 mg/dl or 1.29 mmol/L for women), and elevated LDL-C levels (≥130 mg/dl or 3.36 mmol/L). All trials had a 6-week dietary run-in/lipid therapy washout, a 12-week treatment period, and a 30-day follow-up period.

Patients who completed these trials were eligible to enter a phase III open-label 1-year extension study [25]. A subset of these patients was in turn examined in a phase III, open-label, 2-year extension study [26]. In the extension studies all patients received FA 135 mg/day combined with the moderate dose of the statin to which they had originally been randomized (rosuvastatin 20 mg/day, simvastatin 40 mg/day or atorvastatin 20 mg/day). Their primary purpose was the evaluation of the long-term safety of FA combined with these statins. Additionally, a pooled subgroup analysis of the randomized, double-blind trials was performed in 586 patients with mixed dyslipidemia and type 2 diabetes mellitus (T2DM) [27].

In another 8-week, randomized, double-blind study, the effect on LDL-C levels of the administration of simvastatin 40 mg/day was compared with the fixed combination dosage of FA with rosuvastatin 5, 10 or 20 mg/day, in patients (*n* = 474) with LDL-C ≥160 mg/dl and ≤240 mg/dl and TG ≥150 mg/dl and <400 mg/dl [28].

## Alterations in lipid profiles with FA, statins and FA + statin combinations

The three Phase III trials together enrolled approximately 2,700 patients, of whom 2,575 were available for analysis.

**Fig. 2:**
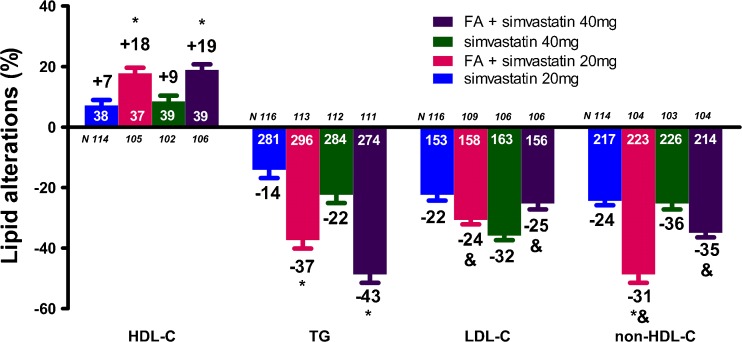
Lipid alterations (%) with the combination of fenofibric acid (FA) 135 mg/day with simvastatin compared with simvastatin alone [22]. * *p* < 0.05 vs. corresponding statin dose. ^&^ *p* < 0.05 vs. FA monotherapy. Bars represent mean ± SEM. The numbers with white color represent the baseline values, whereas the numbers with black color represent the percent changes. HDL-C = high-density lipoprotein cholesterol, TG = triglycerides, LDL-C = low-density lipoprotein cholesterol

The FA/simvastatin study randomized 657 patients of whom 621 were available for analysis [22]. FA + simvastatin 20 mg/day produced a greater increase in HDL-C (17.8 % vs. 7.2 %, *p* < 0.001) and a greater decrease in TG levels (−37.4 % vs. −14.2 %, *p* < 0.001) and very low-density lipoprotein cholesterol (VLDL-C) levels (−38.9 % vs. −19.2 %, *p* < 0.01) compared with simvastatin 20 mg/day (Fig. 2). In addition, FA + simvastatin 20 mg/day resulted in a greater LDL-C decrease compared with FA monotherapy (−24.0 % vs. −4.0 %, *p* < 0.001). Similarly, FA + simvastatin 40 mg/day produced a greater increase in HDL-C (18.9 % vs. 8.5 %, *p* < 0.001) and a greater decrease in TG levels (−42.7 % vs. −22.4 %, *p* < 0.001) and VLDL-C (−51.1 % vs. −35.7 %, *p* < 0.01) compared with simvastatin 40 mg/day, as well as a greater LDL-C decrease compared with FA monotherapy (−25.3 % vs. −4.0 %, *p* < 0.001) (Fig. 2). FA + simvastatin 20 mg/day resulted in significantly greater reductions in non-HDL-C, VLDL-C, total cholesterol (TC) and apoB compared with simvastatin 20 mg/day monotherapy (*p* ≤ 0.012). FA + simvastatin 40 mg/day resulted in similar reductions in non-HDL-C, apoB, TC, and hsCRP compared with simvastatin 40 mg/day [22].

**Fig. 3:**
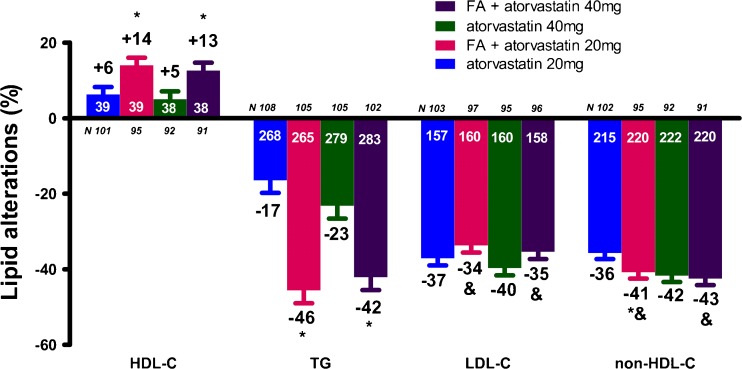
Lipid alterations (%) with the combination of fenofibric acid (FA) 135 mg/day with atorvastatin compared with atorvastatin alone [23]. *p < 0.05 vs. corresponding statin dose. ^&^ *p* < 0.05 vs. FA monotherapy. Bars represent mean ± SEM. The numbers with white color represent the baseline values, whereas the numbers with black color represent the percent changes. HDL-C = high-density lipoprotein cholesterol, TG = triglycerides, LDL-C = low-density lipoprotein cholesterol

The FA/atorvastatin study randomized 613 patients of whom 577 were available for analysis [23]. FA + atorvastatin 40 mg/day combination resulted in significantly greater improvements in TG (−42.1 % vs. −23.2 %, *p* < 0.001), VLDL-C (−53.5 % vs. −35.6 %, *p* < 0.001) and HDL-C levels (12.6 % vs. 5.3 %, *p* = 0.01) compared with atorvastatin 40 mg/day monotherapy, as well as a greater decrease in LDL-C concentration compared with FA monotherapy (−35.4 % vs. −3.4 %, *p* < 0.001, Fig. 3). Similar results were shown when FA + atorvastatin 20 mg/day was compared with FA or atorvastatin 20 mg/day monotherapy. Treatment with FA + atorvastatin 20 mg/day resulted in significantly greater improvements in non-HDL-C compared with FA (*p* < 0.001) and atorvastatin 20 mg/day monotherapies (*p* < 0.05, Fig. 3) [23].

**Fig. 4:**
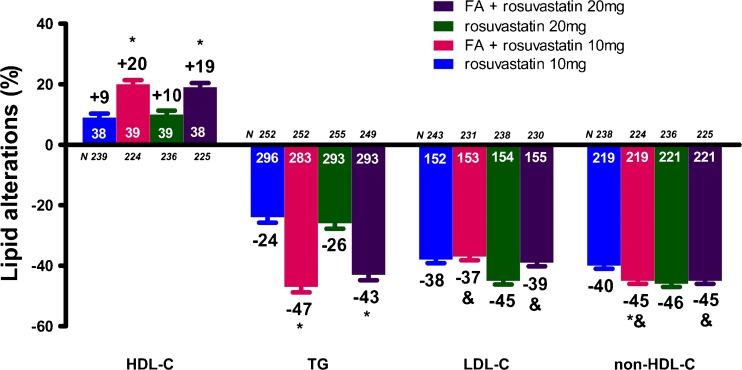
Lipid alterations (%) with the combination of fenofibric acid (FA) 135 mg/day with rosuvastatin compared with rosuvastatin alone [24]. *p < 0.05 vs. corresponding statin dose. ^&^ *p* < 0.05 vs. FA monotherapy. Bars represent mean ± SEM. The numbers with white color represent the baseline values, whereas the numbers with black color represent the percent changes. HDL-C = high-density lipoprotein cholesterol, TG = triglycerides, LDL-C = low-density lipoprotein cholesterol

The FA/rosuvastatin study randomized 1,455 patients of whom 1,377 were available for analysis [24]. FA + rosuvastatin 20 mg/day resulted in a significantly greater increase in HDL-C (19.0 % vs. 10.3 %, *p* < 0.001) and a significantly greater decrease in TG (−42.9 % vs. −25.6 %, *p* < 0.001) and VLDL-C (−50.6 % vs. −42.1 %, *p* = 0.038) levels compared with rosuvastatin 20 mg/day monotherapy (Fig. 4). Furthermore, FA + rosuvastatin 20 mg/day produced a significantly greater LDL-C reduction (−38.8 % vs. −6.5 %, *p* < 0.001) compared with FA monotherapy. Similar results were observed when FA + rosuvastatin 10 mg/day was compared with rosuvastatin 10 mg/day or with FA monotherapy (Fig. 4). FA + rosuvastatin 10 mg/day resulted in significantly greater improvements in non-HDL-C compared with FA (*p* < 0.001) and rosuvastatin 10 mg/day monotherapy (*p* < 0.001), as well as in hsCRP (*p* = 0.013) and VLDL-C (*p* < 0.001) compared with rosuvastatin 10 mg/day monotherapy. FA + rosuvastatin 20 mg/day resulted in a greater improvement in hsCRP levels compared with rosuvastatin 20 mg/day monotherapy (*p* = 0.01) [24].

In another Phase III, multicenter, randomized, double-blind study, rosuvastatin 5 mg/day was administered with FA 135 mg/day in patients with mixed dyslipidemia (*n* = 758) for 12 weeks [29]. Combination treatment resulted in significantly greater improvements in plasma concentration of HDL-C (23.0 % vs. 12.4 %, *p* < 0.001) and TG (−40.3 % vs. −17.5 %, *p* < 0.001) compared with rosuvastatin monotherapy, as well as of LDL-C (−28.7 % vs. −4.1 %, *p* < 0.001) compared with FA monotherapy [29].

A recent trial randomized patients to receive fixed-dose combinations of FA 135 mg/day with rosuvastatin 5, 10 or 20 mg/day or to monotherapy with simvastatin 40 mg/day [28]. The combinations resulted in significantly greater decreases in plasma levels of LDL-C, non-HDL-C, apoB, TG, hsCRP, VLDL-C, TC, and apoC-III, and a significantly greater increase in HDL-C concentration, compared with simvastatin 40 mg/day. For example the reductions in LDL-C levels with FA + rosuvastatin 5, 10 and 20 mg/day were 38.9 %, 46.0 % and 47.2 % respectively compared with 32.8 % for simvastatin 40 mg/day (*p* < 0.01). Significantly higher proportions of patients in each FA/rosuvastatin group achieved optimal levels for LDL-C (<100 mg/dl, *p* < 0.001), non-HDL-C (<130 mg/dl, *p* < 0.001), apoB (<90 mg/dl, *p* ≤ 0.02), and TG (<150 mg/dl, *p* < 0.001) compared with simvastatin 40 mg/day monotherapy. Furthermore, significantly higher proportions of patients treated with each of the FA/rosuvastatin doses simultaneously achieved optimal LDL-C and non-HDL-C levels, as well as optimal levels for all five parameters (LDL-C, non-HDL-C, apoB, HDL-C, and TG), compared with simvastatin 40 mg/day [28].

In a recent 12-week double-blind study, a total of 543 patients with TG ≥150 mg/dl and <400 mg/dl, HDL-C <40 mg/dl (<50 mg/dl for women), and LDL-C ≥130 mg/dl were randomized to FA 135 mg/day or placebo, each co-administered with atorvastatin 40 mg/day + ezetimibe 10 mg/day (Atorva/Eze) [30]. Treatment with FA + Atorva/Eze resulted in a significantly greater improvement in HDL-C (13.0 % vs. 4.2 %, *p* < 0.001) and TG levels (−57.3 % vs. −39.7 %, *p* < 0.001) compared with Atorva/Eze. Both groups experienced a >50 % reduction in LDL-C concentration (−52.9 % with FA + Atorva/Eze, −52.0 % with Atorva/Eze). Furthermore, FA + Atorva/Eze resulted in a significantly greater effect on non-HDL-C, apoB, apoA-I, apoC-III, VLDL-C, and hsCRP compared with Atorva/Eze. More patients in the triple combination achieved the combined target of LDL-C <100 mg/dl, non-HDL-C <130 mg/dl, and apoB <90 mg/dl (88.4 % vs 80.8 % in Atorva/Eze) [30].

## Long-term maintenance of FA-induced metabolic effects

**Fig. 5:**
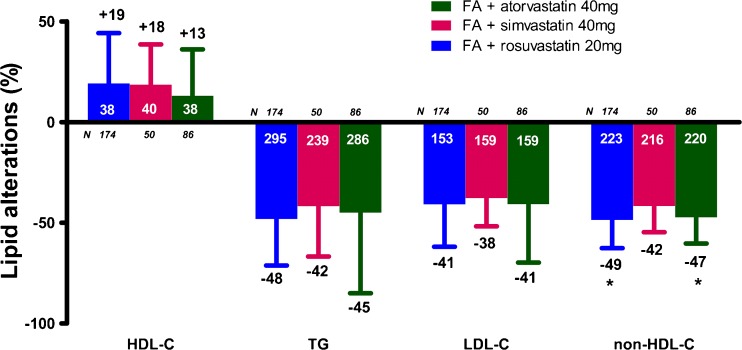
Two-year lipid alterations (%) with the combination of fenofibric acid (FA) 135 mg/day with moderate-dose statin [26]. Bars represent mean ± SD. The numbers with white color represent the baseline values, whereas the numbers with black color represent the percent changes. *p < 0.05 vs. FA + simvastatin. HDL-C = high-density lipoprotein cholesterol, TG = triglycerides, LDL-C = low-density lipoprotein cholesterol

The year 1 extension study reported sustained improvements versus baseline in numerous lipid parameters including TG, HDL-C, LDL-C, apoB, and hsCRP [25]. A total of 310 patients was enrolled and treated in the year 2 extension study, of whom 287 (92.6 %) patients completed the study [26]. The improvements in lipid parameters seen at 1 year were sustained for ≥2 years. Pooled results showed substantial percentage changes in all efficacy variables with no evidence of attenuation of the effects over time [26]. In the pooled year 2 population, the change from baseline to week 116 was +17.4 % in HDL-C, −46.4 % in TG, −40.4 % in LDL-C, −47.3 % in non-HDL-C, −52.8 % in VLDL-C and −37.8 % in TC levels. It should be mentioned that the observed alterations in non-HDL-C, TC and VLDL-C were significantly (*p* < 0.02) smaller in the FA + simvastatin group compared with the FA + atorvastatin or FA + rosuvastatin groups (Fig. 5) [26].

It should be mentioned that the number of patients included in the extension studies is low and it is most likely that highly motivated, good responders were included, a fact that could affect the observed results.

## Time courses and predictors of response

In the FA + statin trials the significant differences in all primary lipid variables between both combination therapies and the corresponding monotherapies were generally observed after 4 weeks of treatment and sustained throughout 12 weeks [22–24]. Furthermore, the effect of treatment was different between certain subgroups of patients. For example, in the subgroup of patients with baseline LDL-C >160 mg/dl (*n* = 247) the decreases in LDL-C were similar in the FA + simvastatin 20 and 40 mg/day (−33.2 % and −34.4 %,) and in the corresponding simvastatin 20 mg/day (−29.0 %) and 40 mg/day (−34.9 %) monotherapy groups. FA + simvastatin (20 or 40 mg/day) had a greater treatment effect on LDL-C levels in women (−29.7 % and −30.3 %, respectively) compared with men (−19.0 % and −20.5 %, respectively) [22].

## Response to FA in patient subgroups

In a recent post-hoc analysis of the three 12-week trials, the administration of FA + low- or moderate-dose statin in approximately 2,000 subjects with the metabolic syndrome was examined [31]. FA + low or moderate-dose statin reduced the number of patients meeting the American Heart Association/National Heart, Lung and Blood Institute (AHA/NHLBI) diagnostic criteria for metabolic syndrome [32] (−35.7 % and −35.9 %, respectively) compared with low-, moderate-, or high-dose statin monotherapy (−15.5 %, −16.6 %, and −13.8 %, respectively) or FA monotherapy (−25.7 %). FA + low- or moderate-dose statin significantly decreased TG (*p* < 0.001) and increased HDL-C (*p* < 0.001) levels compared with the corresponding-dose statin. Interestingly, the prevalence of patients meeting the fasting blood glucose criterion decreased slightly with FA + statin or FA monotherapy but increased slightly with the administration of statin monotherapy. Furthermore, the mean change in fasting glucose was significantly different between FA + low- or moderate-dose statin and low- or moderate-dose statin monotherapy, respectively (*p* ≤ 0.002) [31]. Statins have been associated with a slightly increased risk for T2DM development [33]. The finding that the FA + statin combination attenuates the effects of statin on carbohydrate metabolism parameters needs further investigation.

In patients with mixed dyslipidemia and T2DM at baseline (*n* = 586) the FA + low- or moderate-dose statin combination significantly (*p* < 0.05) reduced HDL-C, TG, and VLDL-C compared with the corresponding dose of statin monotherapy [27]. However, the LDL-C reduction, although similar between FA + low-dose statin and low-dose statin monotherapy, was smaller with FA + moderate-dose statin compared with moderate-dose statin monotherapy (−32.6 % vs. −41.5 %, *p* < 0.01). It should be noted that in the subgroup with baseline LDL-C >160 mg/dl, the LDL-C reductions were similar with FA + low- or moderate dose statin (−45.5 % and −43.5 %, respectively) compared with those observed with low- and moderate-dose statin monotherapy (−38.5 % and −47.1 %, respectively). Furthermore, the reductions in apoB and hsCRP plasma levels were similar between FA + low- or moderate- dose statin and the corresponding statin dose. In this population, it was also shown that the mean changes in fasting blood glucose levels with FA + low- or moderate-dose statin combination were significantly smaller (*p* = 0.038 and *p* = 0.040, respectively) compared with the corresponding dose of statin monotherapy, with which mean fasting glucose levels increased [27].

In the 2-year extension study, the subgroup of patients with T2DM experienced improvement (similar with overall population) in HDL-C (+15.0 %), TG (−42.1 %), LDL-C (−41.8 %), non-HDL-C (−48.2 %), TC (−39.4 %) and VLDL-C (−54.6 %) levels, that was evident up to week 116 [26].

Data concerning the effect of hypolipidemic treatment in elderly subjects is limited. A recent post-hoc analysis evaluated data from patients aged ≥65 years (*n* = 401) with mixed dyslipidemia who received either monotherapy with rosuvastatin 5, 10, or 20 mg/day or FA 135 mg/day, or combination therapy with rosuvastatin (5, 10, or 20 mg/day) + FA 135 mg/day, for 12 weeks in two randomized controlled trials [34]. Each dose of combination treatment decreased significantly the LDL-C concentration compared with FA monotherapy, as well as increased significantly HDL-C and decreased TG levels, compared with corresponding doses of rosuvastatin monotherapy (*p* < 0.001 for all comparisons). LDL-C levels were not significantly different between FA + rosuvastatin and the corresponding dose of rosuvastatin monotherapy. The changes in LDL-C, HDL-C, and TG in the subgroup (*n* = 135) of elderly patients with T2DM in this study were similar to those seen in the overall population [34].

## Safety and tolerability of FA + statin combination

Fibrates are generally safe and well tolerated. The most frequent adverse events (AEs), which are similar to those of statins, are gastrointestinal symptoms (nausea and diarrhea) and musculoskeletal symptoms [myalgia and moderate elevation of creatinine kinase (CK)] [1, 35, 36]. Both fibrates and statins, especially in combination, have been reported to cause myopathy, but the most serious adverse effect, i.e. rhabdomyolysis, is rare if certain precautions are taken [1, 37, 38]. Risk factors for these AEs include renal or hepatic insufficiency, increased age, and several medications [39, 40]. The fenofibrate plus statin combination has been reported to be safer compared with gemfibrozil plus statin combination [41]. In The Fenofibrate Intervention and Event Lowering in Diabetes (FIELD) study, no cases of rhabdomyolysis were described among approximately 900 patients receiving fenofibrate plus a statin [42]. Fenofibrate may also increase creatinine and homocysteine plasma levels [1, 43–48].

The safety of the newer formulation of FA, alone and in combination with low- and moderate- dose statin, was evaluated in the phase III clinical studies (Table 1). The results indicate a similar AE profile between the different FA + statin combination treatments. Furthermore, when the safety profile was examined according to the presence or not of metabolic syndrome, or in patients with T2DM, the AEs were similar with those observed in the overall population [27, 31]. Additionally, the long-term safety of FA + statin was tested for up to 2 years [25, 26]. The most common adverse events were headache, upper respiratory tract infection, nasopharyngitis, and back pain, with the incidence of all adverse events being similar across all combination treatment groups [25, 26]. In these studies, no deaths or rhabdomyolysis were reported during 1- or 2-year follow-up [25, 26].

**Table 1:** Summary of adverse events (AEs) recorded during phase III trials of FA with statins

Study	Number of patients	Treatment related AEs (%)	Rate of discontinuation due to AEs (%)	% patients experiencing AE			
				Myalgia	Abnormal liver function tests^a^	Increased creatine kinase^b^	Rhabdo-myolysis
FA and simvastatin [22]	591	23.9	14.6	4.1	0.8	0.5	0
FA 135 mg/day	119	32.8	NR	5.0	4.2	0	0
Simvastatin 20 mg/day	119	16.0	11.8	3.4	0	0	0
FA 135 mg/day + simvastatin 20 mg/day	119	22.7	NR	4.2	0	0.8	0
Simvastatin 40 mg/day	116	24.1	NR	5.2	0	1.7	0
FA 135 mg/day + simvastatin 40 mg/day	118	23.7	NR	2.5	0	0	0
FA and atorvastatin [23]	554	16.0	8.5	4.1	1.1	0.2	0
FA 135 mg/day	112	12.5	7.1	2.7	0	0	0
Atorvastatin 20 mg/day	113	6.2	2.7	4.4	0	0.9	0
FA 135 mg/day + atorvastatin 20 mg/day	110	20.0*	10.9*	1.8	2.7	0	0
Atorvastatin 40 mg/day	109	18.3	11.0	7.3	0	0	0
FA 135 mg/day + atorvastatin 40 mg/day	110	22.7	12.7	4.5	2.7	0	0
FA and rosuvastatin [24]	533	NR	NR	2.6	0.9	0.2	0
FA 135 mg/day	105	NR	NR	1.0	1.9	0	0
Rosuvastatin 10 mg/day	105	16.9	NR	5.7	0	0	0
FA 135 mg/day + rosuvastatin 10 mg/day	106	27.2*	NR	1.9	1.9	1.0	0
Rosuvastatin 20 mg/day	107	NR	NR	1.9	0	0	0
FA 135 mg/day + rosuvastatin 20 mg/day	110	NR	NR	2.7	0.9	0	0
FA and rosuvastatin versus simvastatin alone [28]	474	15.0	4.2	NR	0.7/0.2 (ALT/AST)	0.4	0
Simvastatin 40 mg/day	119	16.8	5.9	NR	0/0	0	0
FA 135 mg/day + rosuvastatin 5 mg/day	118	13.6	5.1	NR	0/0	0	0
FA 135 mg/day + rosuvastatin 10 mg/day	119	14.3	2.5	NR	0.8/0	0	0
FA 135 mg/day + rosuvastatin 20 mg/day	118	15.3	3.4	NR	1.7/0.9	1.7	0

^a^ALT or AST >3× ULN

^b^Creatine kinase >5× ULN

* Significant difference compared to the low-dose statin monotherapy (*p* < 0.05)

*AE*, adverse event; *FA*, fenofibric acid; *ALT*, alanine aminotransferase; *AST*, aspartate aminotransferase; *FA*, fenofibric acid; *ULN*, upper limit of normal; *NR*, not reported

It should be mentioned that even with the triple combination of FA with atorvastatin/ezetimibe there was no significant difference in the rate of serious or treatment-related AEs and the overall incidence of such events was low [30]. Furthermore, in elderly subjects the safety profile of FA + rosuvastatin administration was generally similar with the individual monotherapies [34].

In conclusion, FA + statin combination treatment did not produce a significant increased rate of serious adverse events compared with monotherapy.

## Clinical implications

Cardiovascular disease (CVD) constitutes the leading cause of death in developed countries. Current treatment guidelines focus on lowering LDL-C as the primary strategy for reducing CVD risk [49, 50]. Statins are associated with a significant CVD risk reduction [51, 52]. However, in the clinical setting a large number of patients treated with lipid-lowering therapies have persistent lipid abnormalities [53–55]. Furthermore, it is now established that patients are still at risk for CVD events, i.e. they have residual CVD risk, even if they are receiving optimal statin treatment [56]. The residual CVD risk remains even if statins are administrated in maximum doses. For example, in the Study of The Effectiveness of Additional Reductions in Cholesterol and Homocysteine (SEARCH), simvastatin 80 mg/day reduced major vascular events by only 6 % compared with simvastatin 20 mg/day [57]. Furthermore, the elevation of the statin dose was associated with an increased risk of myopathy [57]. Residual CVD risk is at least partly explained by the increased levels of TGs and the decreased levels of HDL-C [58, 59]. This risk is enhanced in patients with mixed dyslipidemia, such as T2DM patients or patients with metabolic syndrome, who are characterized by both increased TG levels and decreased HDL-C concentration. Factors which are also implicated, among others, in the increased residual CVD risk in patients with mixed dyslipidemia are the increased levels of the atherogenic small dense LDL particles, of apoC-III and of inflammation-related markers, such as hsCRP and Lp-PLA_2_ [60–64]. A number of interventions have been shown to alter these parameters [65–77]. Fibrates, alone or combined with other drugs, have been demonstrated to reduce plasma levels of the small dense LDL particles and to induce a LDL phenotype modification [1, 3, 78–83]. Furthermore, evidence exists that fibrates improve inflammation-related parameters [10, 83–86]. There is also evidence that fibrates can alter HDL particle distribution, which may play a role in the residual CVD risk [87, 88].

The fibrate-statin combination may substantially reduce the residual CVD risk in certain populations. For example, the Action to Control Cardiovascular Risk in Diabetes (ACCORD) Lipid study included 5,518 patients with T2DM treated with open-label simvastatin, who were randomised to receive either masked fenofibrate or placebo for 4.7 years [89]. The annual rate of the primary outcome (first occurrence of nonfatal myocardial infarction, nonfatal stroke, or death from CVD causes) was 2.2 % in the fenofibrate group and 2.4 % in the placebo group [hazard ratio in the fenofibrate group, 0.92; 95 % confidence interval (CI), 0.79–1.08; *p* = 0.32)]. The annual death rate was 1.5 % in the fenofibrate group and 1.6 % in the placebo group (hazard ratio, 0.91; 95 % CI, 0.75–1.10; *p* = 0.33) [89]. However, in a pre-specified analysis, a benefit regarding the primary outcome rate (first occurrence of nonfatal myocardial infarction, nonfatal stroke, or death from CVD causes) for patients with both a high baseline TG level (≥204 mg/dl) and a low HDL-C baseline level (≤34 mg/dl) was recognized for those on the simvastatin + fenofibrate combination [89]. In fact, this benefit was seen with no evidence of increased adverse events. In the ACCORD Lipid study, elevations of CK ≥10 times the upper limit of the normal range were similar in the fenofibrate group (0.4 %) and in the placebo group (0.3 %, *p* = 0.83) and the same rate of any myopathy/myositis/rhabdomyolysis was reported in the 2 treatment groups (0.1 %) [89].

The evidence derived from randomized trials favors the use of FA combined with a low- or moderate-dose statin for the reduction of the residual CVD risk. This combination improves all primary lipid variables. For example, the percentage of patients simultaneously achieving LDL-C <100 mg/dl, non-HDL-C <130 mg/dl, apoB <90 mg/dl, HDL-C >40 mg/dl (men) or >50 mg/dl (women), and TG <150 mg/dl was ≥5-fold higher with the combination of FA + low-dose statin compared with low-dose statin monotherapy, and approximately 7-fold higher with the combination of FA + moderate-dose statin compared with moderate-dose statin monotherapy (*p* < 0.001 for both) [27]. FA added to statin therapy has also repeatedly been shown to reduce hsCRP levels. This may be of clinical relevance since the reduction of hsCRP levels with rosuvastatin has been associated with CVD event decrease [90]. However, it should be mentioned that there is no clinical outcomes data to show that treating the non-LDL-C variables with FA will reduce major CVD endpoints.
